# The war on drugs is a war on us: young people who use drugs and the fight for harm reduction in the Global South

**DOI:** 10.1186/s12954-023-00914-7

**Published:** 2024-02-17

**Authors:** M-J Stowe, Rita Gatonye, Ishwor Maharjan, Seyi Kehinde, Sidarth Arya, Jorge Herrera Valderrábano, Angela Mcbride, Florian Scheibein, Emmy Kageha Igonya, Danya Fast

**Affiliations:** 1 The Kirby Institute, UNSW Sydney, Wallace Wurth Building, Sydney, NSW 2052 Australia; 2 South African Network of People Who Use Drugs (SANPUD), Cape Town, South Africa; 3 Community Orientated Substance Use Program, University of Pretoria, Pretoria, South Africa; 4 Women in Response to HIV/AIDS and Drug Addiction, Umuahia, Nigeria; 5 Management Center Innsbruck, Innsbruck, Austria; 6 Youth RISE Nigeria, Federal Capital Territory, Nigeria; 7 State Drug Dependence Treatment Centre, Institute of Mental Health, Pt B.D.S University of Health Sciences, Rohtak, India; 8 Ágora, Mexico City, Mexico; 9 School of Health Sciences, South East Technological University, Waterford, Ireland; 10 African Population and Health Research Centre, Nairobi, Kenya; 11 University of British Columbia and British Columbia Centre On Substance Use, Vancouver, Canada

**Keywords:** Young people who use drugs, Global South, Low- and middle-income countries, Drugs, Drug policy, Harm reduction, Human rights

## Abstract

In the Global South, young people who use drugs (YPWUD) are exposed to multiple interconnected social and health harms, with many low- and middle-income countries enforcing racist, prohibitionist-based drug policies that generate physical and structural violence. While harm reduction coverage for YPWUD is suboptimal globally, in low- and middle-income countries youth-focused harm reduction programs are particularly lacking. Those that do exist are often powerfully shaped by global health funding regimes that restrict progressive approaches and reach. In this commentary we highlight the efforts of young people, activists, allies, and organisations across some Global South settings to enact programs such as those focused on peer-to-peer information sharing and advocacy, overdose monitoring and response, and drug checking. We draw on our experiential knowledge and expertise to identify and discuss key challenges, opportunities, and recommendations for youth harm reduction movements, programs and practices in low- to middle-income countries and beyond, focusing on the need for youth-driven interventions. We conclude this commentary with several calls to action to advance harm reduction for YPWUD within and across Global South settings.

## Introduction

Across the Global South, young people are often exposed to multiple interconnected health and social harms because of the war on drugs, with many low- and middle-income countries imposing racist, classist, and prohibitionist drug policies through a continuum of violence that encompasses physical and structural assaults [1–4]. Young people who use drugs (YPWUD) in the context of various intersections of age, race, class, gender, sexuality, mental health challenges, and involvement in criminalized income generation activities such as sex work often experience heightened violence and oppression and worse health and social outcomes [5–10]. Despite increasing global coverage of harm reduction services [11], there remains a lack of youth-focused harm reduction programs, especially in low- and middle-income countries [3, 12, 13]. In general, across these settings public health systems are often characterized by systemic under investment and deteriorating infrastructures, human resource crises, and corruption [14]. Conflicting public health, international donor funding, and law enforcement agendas impede the implementation of evidence-based harm reduction programs. The criminalization and moralization of drugs and the people who use them powerfully undermine access to those harm reduction programs that do exist [2, 15, 16]. In places where it is possible to access harm reduction programs (usually run by non-governmental organizations), YPWUD are disproportionately underserved relative to older populations in these contexts [3, 17, 18].

Particularly in the Global South, the war on drugs is too often a violent, racist, and classist war on YPWUD in the context of multiple and intersecting forms of oppression and limited access to care [19, 20]. Yet, YPWUD as well as youth-led and youth-inclusive organizations in these settings are actively pioneering harm reduction programs to meet their needs and the needs of their communities. This commentary is authored by YPWUD—past and present—from countries throughout the Global South, alongside academic and community allies from low- and middle-income countries as well as higher income countries. We are a heterogeneous group, yet each of us embraces harm reduction as a set of ideological principles and pragmatic strategies rooted in social and health justice and a commitment to human rights, including the right to health for all people [21]. We believe that harm reduction is characterised by an absence of judgement towards drug use and respect for an individual’s choice to use drugs [22]. Since 2009, the World Health Organisation has provided a list of harm reduction interventions for the prevention, treatment and care of people who inject drugs and are living with or at risk of HIV, including needle distribution and exchange programs, opioid agonist therapies, and HIV testing and treatment programs [23]. However, we argue that harm reduction programs for people who use drugs, including YPWUD, must extend beyond HIV prevention, testing and treatment. Programs must include interventions such as the distribution of a range of supplies (not just needles—for example, safer smoking kits), take-home naloxone (the opioid overdose antidote) programs, drug checking services, drug consumption spaces, and peer-led information sharing, support, and advocacy. Unfortunately, across many Global South settings, donor funding has been insufficient to support this kind of comprehensive harm reduction programming, including for YPWUD [3, 12–14]. We have therefore taken matters into our own hands, in some cases despite tremendous risks to our safety and the safety of our communities.

The purpose of this commentary is to further ignite much needed conversations about YPWUD and harm reduction in the Global South. It centres the diverse efforts of young people, activists, allies, and organisations across numerous Global South settings to enact programs such as those focused on peer-to-peer information sharing and advocacy, overdose monitoring and response, and drug checking. This is by no means a comprehensive overview of what is happening when it comes to YPWUD and harm reduction in the Global South. So many success stories are missing from what follows, in part because we struggled to connect—and stay connected with—young drug user activists and harm reduction practitioners across the globe. These young people are oftentimes overworked, overwhelmed, and under compensated as they attempt to keep themselves and their communities safe while under the weight of poverty, the drug war, and other forms of oppression. They may be completely new to these kinds of scholarly outputs and lack access to mentors who are able assist with the challenging work of writing (often in a second language). The timeline of this kind of work can also be frustrating; a large investment of time and energy is required, but the rewards of contributing are often unclear, especially as time passes and a publication has still not come to fruition. Sharing several of the harm reduction success stories that we were able to collect, we draw on our experiential knowledge and expertise to identify and discuss key challenges, opportunities, and recommendations for youth harm movements, programs and practices in low- to middle-income countries and beyond, focusing on the need for youth-driven interventions.

## A note on language

There are major limitations to the language of both “young people who use drugs (YPWUD)” and the “Global South” that we employ throughout this commentary. Both terms may ultimately obscure more than they reveal, because they often seem to place finite boundaries around what in reality are much messier social and geographic categories. Definitions of “young people” and “youth”—and lived experiences of these categories—vary widely across settings and contexts, where intersections of age, gender, sexual orientation, class, race, human immunodeficiency virus (HIV) status, and other dimensions of positionality are mediated by configurations of power and political economy to shape these social categories and lived experiences. Does it make sense to talk about young people and youth as those under twenty-five or thirty years of age, when many individuals continue to strongly identify with these categories—and with youth drug user activism and movements—well into their thirties, often due to entrenched, shared circumstances of precarity as well as shared visions for possible solutions [24, 25]? Conversely, do age-definitions of young people and youth make sense when referring to those for whom poverty, lack of education, unemployment, violence, migration, HIV, and other difficulties have forced them to move directly from childhood to adulthood, without the possibility of experiencing youth as a period of transition, activism, and power [26]?

Similarly, it may not make sense to talk about a Global South composed of countries and regions in Africa, Latin America, and parts of Asia that meet certain criteria according to the World Bank income-per-capita index, when many so-called “Global North” settings are home to populations experiencing similar levels of entrenched poverty and structural oppression, also as a result of historical and ongoing processes of colonialism and capitalism [27]. Recognizing the limitations of this language, in this commentary we have chosen to use the term Global South (rather than listing out discrete countries and regions in various instances) in order to emphasize some of the common and disproportionate impacts of the war on drugs on YPWUD across low- and middle-income countries. We use the term young people to mark some of ourselves out as a unique demographic with specific harm reduction priorities, needs, and desires that is simultaneously characterized by fluidity of meaning and association not necessarily determined by numerical age.

## Shared challenges

There is a severe lack of data about YPWUD in the Global South [28]. What we do know is that those between the ages of 14 and 34 account for more than one third of the population in low-income countries, and rates of substance use are high and climbing among this age range [29, 30]. YPWUD in the Global South are vulnerable to a myriad of health and social harms (exacerbated by the COVID-19 pandemic), including blood-borne infections (e.g., HIV, hepatitis C), fatal and non-fatal opioid involved overdoses, skin and soft tissue infections, police violence and extrajudicial murder, and mass incarceration [9, 30–32].

Global coverage of harm reduction programs is suboptimal, but this is particularly the case in lower- and middle-income countries [11]. For example, the Global State of Harm Reduction Report [33] highlights that only fourteen out of twenty-five countries or regions in these parts of the world have existing needle exchange programs. Countries or regions that do have needle exchange programs generally also provide access to opioid agonist therapies such as methadone, although coverage is often low. However, even in settings with needle exchange and opioid agonist therapy programs, life-saving interventions such as drug consumption spaces (also called overdose prevention and supervised injection sites), drug checking services, and take-home naloxone programs remain largely unavailable [11].

Across Global South settings, YPWUD and the organizations they are a part of face particularly dire challenges in implementing much needed harm reduction programs due to hostile and militarized governments, violent policing, punitive laws and forced treatment and rehabilitation models, precarious and conditional state and international funding, systemic corruption and entrenched stigma [13, 34]. High levels of unemployment, poverty and homelessness often combine with the criminalization of drug use (in some cases via the death penalty and extrajudicial killings), sex work, and sexual and gender identities to produce egregious human rights violations and make harm reduction organising and action difficult if not impossible [26, 35, 36]. In a system of prohibition, the Global South is also disproportionately affected by the various negative effects of the global demand for illicit drugs, which particularly impacts countries in Latin America, South East Asia, the Middle East and North Africa, as well as in transit regions such as West Africa [2, 37, 38].

## Success stories

While significant challenges remain for implementing comprehensive harm reduction programming for YPWUD across Global South settings, several of us are actively involved in implementing various harm reduction programs in our countries. We share these examples here with the goal of inspiring youth drug user activism and meaningful policy and programming change across lower- and middle-income settings globally.

### Empowering young women who use drugs in East Africa through online networking and peer support

A majority of empowerment efforts directed towards women who use drugs are under-resourced, patriarchal, and fail to consider how complex intersections of age, gender, class, culture, and geography shape drug use [17]. In 2022, the community-led organization Women in Response to HIV/AIDS and Drug Addiction (WRADA) set out to build a network of young women who use drugs in Kenya, Uganda and Tanzania with support from the International Network of People who Use Drugs. This multi-year community initiative involves bi-monthly online peer support forums using Microsoft Zoom. Young women meet on Zoom to discuss and document regional harm reduction challenges and emerging trends in drug use and sex work and develop sexual and reproductive health and harm reduction information tailored to their communities. The project fosters empowerment by increasing the knowledge, skills and capacities of participants, growing grassroots harm reduction research and advocacy, developing context-driven approaches to promoting human rights, and providing peer mental health support. Despite challenges with internet connectivity and technological know-how among participants, the project has resulted in improved relationships between young women who use drugs and local harm reduction programs across East Africa, greater advocacy for adoption of best practices through a peer-to-peer learning model, the generation of more robust evidence for regional harm reduction and drug policy reform, and increased visibility of young women who use drugs in the region.

### Distributing take-home naloxone kits and overdose education in South Africa

In South Africa, YPWUD are an underserved population frequently exposed to the health and social harms of HIV and hepatitis C, skin and soft-tissue infections, poverty, unstable housing and homelessness, and multiple forms of physical and structural violence [26, 39]. Heroin use and overdose are increasingly common among youth [40]. While South Africa’s essential medicines list includes naloxone for the management of overdose, to date no state-sponsored naloxone distribution programs exist. To improve access to this lifesaving overdose antidote among YPWUD and others, in 2021 the South African Network of People Who Use Drugs (SANPUD) piloted the country’s first (and to date only) take-home naloxone (THN) program. The program was piloted in Cape Town, Tshwane, and eThekwini, with two workshops held in each city over a two-week period. It involved peer-delivered overdose education, including practical training on the administration of naloxone. Participants received a naloxone kit with four ampoules of naloxone (0.4 mg/1 ml) and required medical equipment, as well as a step-by-step guide to responding to and managing opioid overdoses. The design of these materials was informed by several community advisory groups that included youth-led and -focused groups. During the pilot, three opioid overdoses were successfully reversed. Unfortunately, lack of state funding and political buy-in has halted the continuation and expansion of the program. However, the success of the pilot represents an important moment in the fight against racist and violent drug policies that continue to criminalize and disproportionately burden YPWUD, and in particular Black and Brown YPWUD. The successful co-design and delivery of a youth-led THN program underscores growing calls for non-restrictive state and NGO funding that can be used to support evidence-based interventions beyond a narrow set of prescriptive interventions and programs (e.g., HIV prevention programs focused on people who inject drugs).

### Training frontline workers to provide harm reduction services to young people who inject drugs in Nepal

In 2019, a government survey revealed that there are over 130,000 people who use drugs in Nepal, with young people (defined as < 30 years of age) accounting for more than two-thirds of this figure [41]. Young people who inject drugs in this setting are disproportionately vulnerable to various health, social, and legal harms [42, 43]. In response, in 2022 five community organizations (YKP Lead, Sathi Samuha, Recovering Nepal, Youth RISE, and Youth LEAD) came together to pilot a new training and service delivery project to better address the needs of young people who inject drugs. As a first step, focus group discussions with youth and in-depth interviews with service providers led to the identification of shared problems and possible healthcare and harm reduction-oriented solutions, including the identification of key areas for outreach throughout Kathmandu Valley. Using this information, twenty-two service providers from organizations providing harm reduction services in Kathmandu Valley were trained to better understand how social, cultural, political, economic, geographic and technological contexts affect the practices and service needs of young people who inject drugs in diverse communities. Context-informed approaches to care were developed, including online and peer-to-peer outreach and counselling, expanded hours of operation for on-the-ground harm reduction services, and better referral mechanisms among providers. The findings and recommendations generated by the pilot project were later shared with a national audience of providers and organizations. The pilot project enhanced awareness and knowledge among providers and better equipped them with the skills they need to adapt and deliver harm reduction to young people who inject drugs.

### Exchanging knowledge, building advocacy, and challenging punitive drug policies among YPWUD in Mexico

In Mexico, young people are increasingly exposed to the negative impacts of the country’s militarized and violent approach to addressing drug use and trafficking, resulting in regular human rights violations [44, 45]. International donor funding continues to support “tough on crime” rhetoric and prioritise law enforcement interventions over evidenced-based healthcare and harm reduction approaches. In response, YPWUD have mobilised to hold the now annual Support Don’t Punish Festival as a means of regularly engaging with each other, sharing harm reduction knowledge and challenging punitive drug policies. The festival is held each year on June 26th as a community response to the United Nations International Day against Drug Abuse and Illicit Trafficking. It is delivered by Instituto RIA and Reverde Ser Colectivo and provides a safe, non-judgemental space for young people to fight for their human rights. Festival activities include youth-led marches, harm reduction information booths, showcases of youth entrepreneurship, and performances by bands opposed to the oppression of YPWUD. During the COVID-19 pandemic, the festival transitioned to a virtual event, expanding its reach to include multiple countries. Throughout the rest of the year, the festival supports other activities, such as the creation of collective murals, art exhibits, social media content, and harm reduction information materials, including entertaining videos promoting drug use best practices. The Support Don’t Punish Festival has become a vital platform for harm reduction knowledge exchange, advocacy and challenging punitive drug policies among YPWUD in Mexico and beyond.

### Drug checking services in Colombia

Drug checking services in Colombia have been implemented to decrease exposure to the growing harms associated with an unregulated drug supply. In general, drug checking services have multiple aims: to conduct chemical analyses of substances submitted directly by the public; to return results to service users; to provide a platform for tailored (rather than general) information exchange between service users and services; and to ultimately reduce harms [46]. Although reducing drug-related harm via changes in drug using practices at the point of consumption is key to the success of drug checking, these services are also highly valued for generating real-time information about drug market trends that can be actioned rapidly via text message and social media alerts [47].

With volunteers and harm reduction experts located in Bogotá, Medellin, and Cali, the non-governmental organization Acción Técnica Social implemented a drug checking service beginning in 2013 through a project entitled Échele Cabeza Cuando se de en la Cabeza (EC; translated as Use Your Head Before It Goes to Your Head) [48]. Since its inception, EC has involved YPWUD and used innovative harm reduction communication strategies to promote self- and community-care: protests, street art, posters, handouts, flyers, videos, memes, and maintaining a significant presence on social media. EC began by offering drug checking services on-site at raves, festivals, and nightlife events; in 2016, fixed-site drug checking services were introduced [48]. Wherever drug checking services are provided, YPWUD are provided with tailored information and support backed by scientific evidence. Based on the testing done across these settings, EC regularly posts to social media about substances, test results, and alerts, supporting real time dissemination of critical harm reduction information. EC has demonstrated the importance of monitoring the drug market and building online and in-person networks of people who use drugs, including YPWUD. Unfortunately, despite the effectiveness of this program and some support from the Mayor’s Office of Bogotá and the Columbian Drug Observatory, financial restrictions continue to limit the reach of the program [48].

## Calls to action

Building on our discussion of shared challenges and success stories, we conclude by putting forward eight calls to action to advance harm reduction for YPWUD across Global South settings:

1. The global war on drugs is a failure with enormous health, social, and human rights costs for YPWUD in Global South settings. The decriminalization and demilitarization of drug use is foundational to improving health and social outcomes among YPWUD, and in particular those experiencing oppression along multiple axes of age, gender, sexual orientation, class, race, and HIV status.
2. YPWUD from across the Global South must be meaningfully involved in harm reduction policy, programming, and activism. We should be at the table with government (when this is possible) as well as non-governmental organizations, donors, and academic institutions when decisions are being made. All of the success stories detailed above demonstrate that our participation is essential to the development of tailored, context-responsive, and effective services and programs that promote equity and uphold human rights.
3. Governments and non-governmental organizations in low- and middle-income countries should regularly collect accurate data on drug use patterns, including patterns among YPWUD, and use that data to inform harm reduction policy and programming. For example, at present, the Government of Nepal only collects data once every five to six years, limiting the relevance of this data to policy and practice. Yet, the pilot project described above demonstrates how up to date information gleaned through focus groups and in-depth interviews facilitates the adaptation and development of effective harm reduction programs and practices.
4. Financial resources, including international donor and domestic funding, must be shifted from punitive law enforcement and drug supply reduction approaches towards supporting a continuum of community-based, evidence-informed online and on-the-ground harm reduction programs, including peer-to-peer information generating and sharing programs and advocacy networks, take-home naloxone programs, drug checking services, and drug consumption spaces.
5. Government, non-governmental organization, and international donor funding focused on harm reduction should be less tied to a narrow set of interventions—namely, HIV prevention programs focused on people who inject drugs—in order to better support the diverse efforts of YPWUD and youth-led and -inclusive community organizations to meet their harm reduction needs.
6. Too often, promising pilot projects end because of a lack of sustained funding. Government, non-governmental organizations, international donors, and civil society should work to identify and scale up promising pilot projects undertaken by and with YPWUD. The focus of those providing funding should be on what is happening and working on the ground—and online—among YPWUD. It should be recognized that funding these projects often produces better results than campaigns and programs that are overly general and imposed from the top down.
7. Towards this end, there must be better coordination and collaboration between governments, non-governmental organizations, international donors, and civil society.
8. There must be greater efforts to build capacity among YPWUD in the Global South to undertake harm reduction-focused research, including the evaluation of their own programs. Many YPWUD and their mentors have tremendous experiential knowledge regarding drug use and harm reduction programs, practices, and needs in their communities, but lack the ability to translate that knowledge into traditional scholarly outputs, including peer-reviewed publications and grants. We should be able to narrate our own stories and share our experiences and expertise. Those conducting funded research—and building their careers—in Global South settings who do have these skills (namely, many academics based in Northern universities), must commit to doing some of this capacity building work together with YPWUD. This can take the form of a significant time investment, such as co-authoring publications and grants (as the senior author did with this piece). It can also take the form of a financial investment. YPWUD should be adequately compensated for their time and expertise when working on these kinds of projects.

## Data Availability

Not applicable.
